# Editorial: Fluid Therapy in Animals: Physiologic Principles and Contemporary Fluid Resuscitation Considerations

**DOI:** 10.3389/fvets.2021.744080

**Published:** 2021-10-20

**Authors:** William W. Muir, Dez Hughes, Deborah C. Silverstein

**Affiliations:** ^1^College of Veterinary Medicine, Lincoln Memorial University, Harrogate, TN, United States; ^2^Melbourne Veterinary School, Faculty of Veterinary and Agricultural Sciences, University of Melbourne, Melbourne, VIC, Australia; ^3^Department of Clinical Sciences and Advanced Medicine, School of Veterinary Medicine, University of Pennsylvania, Philadelphia, PA, United States

**Keywords:** physiology, fluid compartments, perfusion, volume kinetics, monitoring, novel fluids

## Introduction

What an oversight! Fluids are drugs (1), so why has one of the most administered, and arguably beneficial, therapies employed in veterinary medicine been so inadequately investigated? Intravenous fluids (i.e., drug) can produce positive or negative effects dependent upon their dose and the circumstances (i.e., context) that exist when they are administered (2, 3). Improved understanding of the physiologic principles that determine the effects and consequences of fluid therapy in healthy and diseased animals is essential to good clinical practice (4–86). Notably, most of the “evidence” investigating fluid therapy in animals has been obtained from studies that are not randomized, properly controlled, blinded, adequately powered, or fail to identify predefined primary or secondary outcomes (73, 75, 87, 88). As a result, much of the medical literature provides, “little reliable information on the effectiveness of fluid resuscitation” in diverse clinical scenarios (2, 3, 89–93). Future studies must address these limitations since “fluid therapy might be more difficult than you think” (94), “nothing is more dangerous than conscientious foolishness” (95) and “solely the dose determines that a thing is not a poison” (96). For example, the pharmacokinetics of fluids administered to cats, dogs, horses, or cattle are largely unknown and generally not considered when designing fluid therapy trials although it has been addressed in the human medical literature for more than 20 years (31, 97–100).

This issue of Frontiers in Veterinary Science provides: (1) A review of the terms used to define or describe fluid therapy (Chow); (2) An update on body fluid compartments and the physiological concepts that guide fluid therapy (Stewart; Woodcock and Michel; Smart and Hughes; Cooper and Silverstein); (3) A discussion of fluid kinetics and its relevance to fluid administration in cats (Yiew, Bateman, Hahn, Bersenas, Muir; Yiew, Bateman, Hahn, Bersenas); (4) Contemporary recommendations for the administration of IV fluid regimens in small and large animals (Rudloff and Hopper; Crabtree and Epstein; Adamik and Yozova); (5) The effects of IV fluids on the coagulation system (Boyd et al.); (6) A discussion of fluid administration in animals with naturally occurring disorders and diseases (e.g., food deprivation, dehydration, sepsis, renal, pulmonary, trauma, hemorrhage, traumatic brain injury; Freeman; Dias et al.; Montealegre and Lyons; Constable et al.; Hall and Drobatz; Pigot and Rudloff; Langston and Gordon; Adamantos) including refractory hypotension (Valverde) and cardiopulmonary resuscitation (Fletcher and Boller); (7) The consequences of fluid overload (Hansen); (8) A description of dynamic fluid therapy monitoring techniques (Boysen and Gommeren); (9) An introduction to fluids of the future (Edwards and Hoareau); and (10) Alternative methods for fluid delivery (Gholami et al.). The information and citations contained within this collection serve as a rich resource for the design of future studies investigating the safety and efficacy of intravenous fluid therapy in animals.

## Body Fluid Compartments

Water (i.e., total body water: TBW) is responsible for ~60–70% of body weight (BW) and is the primary component of all body fluids (101). The two main body fluid compartments are the intracellular fluid (ICF) and extracellular fluid (ECF). Approximately two-thirds (≈40%) of TBW is intracellular fluid (ICF) and one-third (≈20%) extracellular fluid (ECF). The ECF is comprised of four sub-compartments, the intravascular fluid volume (i.e., plasma volume: PV; 4–5% BW), the fluid that surrounds cells (i.e., interstitial fluid volume: IFV; ≈15–18% BW), lymph, and fluids contained within epithelial lined spaces (transcellular fluids) [Stewart; (101–104)]. Severe obesity can increase the relative percentage of the ECF by up to 50% of TBW (≈ 30% BW) (105). The intravascular volume (i.e., blood volume: BV) is comprised of the red cell volume (RBCV; 6–8% BW) and plasma volume (PV) (106, 107). If the packed red blood cell volume (PCV) is known BV can be determined (i.e., BV = PV × 100/100-PCV) (106). Transcellular fluids are infrequently considered when determining water and solute requirements in simple stomached animals but become important in horses and ruminants (108). Determination of the body's fluid compartments is technically challenging, time consuming, and often inaccurate (109–111). Substances used for this purpose (i.e., “dilutional tracer technique”) must be non-toxic, easily detectable and sustain a steady state concentration within the compartment (112–114).

Contemporary evidence suggests that the PV is comprised of circulating and non-circulating (15–25% of PV) components, the latter being located within an endothelial surface or glycocalyx layer (GLX) and the channels between vascular epithelial cells (115, 116). The GLX interacts freely with plasma proteins and acts as an surface layer “gatekeeper” for larger molecules selectively reducing plasma solute distribution volume dependent upon their molecular weight (MW), shape (i.e., effective molecular radius), electrical charge, and concentration (11–14, 117). Crystalloids have a shorter intravascular retention time than colloids (100, 118–121).

## Blood Distribution

Blood volume is distributed between the pulmonary (18–20%) and systemic (78–80%) circulations dependent upon their (e.g., brain, heart, lung, and gut) oxygen requirements (VO_2_). Veins are ~30 times more compliant than arteries, contain up to five times more adrenergic receptors than arteries and normally serve as blood reservoirs (122). Some investigators have described the blood volume contained within the systemic veins as either unstressed or stressed (123, 124). The unstressed volume (Vu; ≈70% BV) is equivalent to the blood volume required to fill the veins without increasing the transmural pressure above zero mmHg and the stressed volume (Vs; ≈30% BV) as the volume of blood required to increase the transmural pressure to values above zero (123). Under normal circumstances Vu is believed to serve as a reserve volume that can be mobilized by increasing sympathetic activity (i.e., alpha 1 receptors) thereby increasing vs. (i.e., “effective” BV) (125, 126). The mean circulatory filling pressure (MCFP) is defined as the mean vascular pressure that exists in the systemic circulation after the heart is stopped and is argued to be determinant of venous return and cardiac output (127, 128). A growing number of vascular physiologists however consider this interpretation to be abstract and erroneous opting to believe that cardiac contraction is the independent variable that drives blood flow and determines cardiac output (129–136).

## Water Balance

Water balance (i.e., water intake and output) is governed by a variety of neural and neuroendocrine high-gain homeostatic feedback mechanisms that include, osmoreceptors, osmotically stimulated thirst receptors, hormones [e.g., renin-angiotensin-aldosterone system (RAAS), angiotensin-converting enzyme-2 (ACE2)/angiotensin 1–7 (Ang 1–7), vasopressin (antidiuretic hormone: ADH), erythropoietin (EPO), atrial natriuretic peptide (ANP)] and membrane water channels (i.e., aquaporins), especially those located in the renal tubules (137–144). The kidney is responsible for regulating fluid, electrolyte balance and blood volume (145–147). The kidney also produces and secrets erythropoietin (e.g., low Hb, PaO_2_, flow) signaling bone marrow to produce more red blood cells. Activated atrial stretch receptors secrete ANP producing vasodilation and increases in glomerular filtration, salt and water excretion, and vascular permeability, thereby regulating PV and lowering arterial blood pressure (ABP) (141, 148). Therefore, the kidney is regarded as a key determinant of both PV and BV. Negatively charged glycosaminoglycans (GAGs) located in the interstitial spaces and lymphatics of the skin also function as non-renal regulators of sodium ion concentration and ECF volume (7, 8) serving as indirect controllers of arterial blood pressure (ABP) by shifting fluid from the interstitial to the intravascular space (7, 8, 149).

## Blood Flow and Tissue Perfusion

The heart and vasculature deliver blood to and from the systemic and pulmonary circulations and, in conjunction with interstitial compliance and the lymphatic system, are responsible for ensuring the continuous circulation of fluid throughout the body (5, 5, 150–156). Three categories of capillaries are involved in the exchange of fluid, gases (O_2_, CO_2_), and solutes (e.g., albumin) (155, 157). Non-fenestrated or continuous capillaries nourish the tissues of the nervous system, muscle, connective tissue, skin, lung, and fat. Fenestrated (i.e., contain “pores”) capillaries perfuse the kidneys, intestinal mucosa, synovial linings, exocrine glands and sinusoidal or discontinuous capillaries with large intercellular breaks (i.e., pores) filter blood in the liver, spleen, and bone marrow (11). All three are coated to a greater or lesser extent by the semi-permeable negatively charged GLX [(11–13); [Yiew, Bateman, Hahn, Bersenas; Rudloff and Hopper; Crabtree and Epstein; Adamik and Yozova; (155, 158)]. Plasma filtration among the different types of capillaries is determined by hydrostatic (mmHg) and osmotic (mOsm/L) pressures, the number and size or their fenestrations [i.e., “pores”]), capillary surface area, the thickness of the GLX, the pre- to postcapillary vascular tone (i.e., resistance ratio), and tissue compliance (3, 159–162). Capillaries in the renal glomeruli are fenestrated (pore: 30–60 nm) but have a smaller effective pore size (pore: ≤ 15 nm) due to the influence of the GLX on the filtration of larger (>40–50 kDa) molecules (163, 164). Non-fenestrated capillaries (e.g., central nervous system blood brain barrier; ≤ 1–2 nm) with numerous endothelial transport vesicles enable transcytosis (i.e., transcellular transport of macromolecules). They are less permeable to fluid and electrolyte exchange than fenestrated capillaries, although water and small solutes pass through endothelial intercellular clefts in accordance with hydrostatic pressure differences (157). Non-fenestrated “continuous” capillaries (e.g., skin, lungs, and the blood-brain barrier) have a comparatively small effective pore size (pore: 3–5 nm) that inhibits the trans-vascular flux of fluid and most solutes (160, 163–165).

The GLX constitutes ~2% of the PV and functions as two layers: a less permeable, dense branch-like inner layer composed of heparin sulfate and glycoproteins and a more permeable porous outer later composed of plasma proteins and glycosaminoglycans (13, 104, 166). The GLX limits albumin (i.e., large molecule) and RBC access, leukocyte contact with the inner layer and endothelial surface (13, 104, 166), participates in cell signaling (i.e., nitric oxide-induced vasorelaxation), provides anti-coagulant effects and protects endothelial cells from oxidative stress (107). Small molecules, such as water, gases, small lipids, and lipid-soluble molecules diffuse freely through the GLX through endothelial intercellular clefts or by facilitated diffusion (158). Larger molecules (i.e., colloids) negligibly penetrate the GLX and distribute in a smaller intravascular volume than crystalloids which readily distribute throughout the entire intravascular space. Recent studies suggest that crystalloid-to-colloid ratios should range from 0.7 to 1.4:1 in contrast to older ratios (i.e., 1:3) (167–175) and that crystalloid-to-blood ratios > 1:1 produce perivascular edema, pulmonary parenchymal stiffness (176), impaired coagulation [Boyd et al.; (177, 178)], increased blood loss (44), and increased vasopressor requirements (43). Disagreements favoring colloids over crystalloids rest more on their delayed diffusion than on their safety [(44, 50–53); Boyd et al.; (179)], risk-benefit ratio (Adamik and Yozova) or cost.

## Transvascular Fluid Flux

### Traditional Theory

The dynamics of fluid flux (J_v_) across capillary walls is historically attributed to Earnest Starling's observations of fluid absorption from connective tissue spaces (Starling 1896) (180). He concluded that capillary hydrostatic pressure was responsible for transudation of a small amount of fluid into the tissues (“frictional resistance of the capillary wall”), thereby forming lymph, and that the colloid osmotic pressure produced by plasma proteins was responsible for fluid absorption. He also postulated that the forces moving fluid in and out of the capillary were almost balanced. Subsequent experiments resulted in mathematical descriptions of Starling's hypothesis and suggested equations wherein J_v_ (i.e., transvascular fluid flux) is a balance of intravascular capillary (c) intravascular and interstitial (i) hydraulic (i.e., hydrostatic pressure: P) and oncotic [π: colloid osmotic pressure (COP)] forces (Kedem–Katchalski equations) (181). Capillary hydrostatic pressure (P_c_) is a function of the hydrostatic P from the inflow (arterial: a) to the outflow (venous: v) end of the capillary and are dependent upon the pre- and post-capillary resistances (R), assuming blood flow remains constant (182–186). A decrease in Ra (e.g., arteriolar vasodilation) or an increase in Rv (venoconstriction) decreases Ra/Rv and increases both P_c_ and Jv (3). Under normal circumstances Pc is more sensitive to changes in Pv than Pa but during intense arterial vasoconstriction, Pc decreases rapidly (increased Ra/Rv) (3, 185). Plasma proteins are responsible for generating π_c_ and COP is the hydrostatic pressure required to prevent fluid movement into the plasma or, alternatively, the pressure that pulls fluid across the capillary wall into the plasma. Capillary P_c_ (i.e., hydraulic push) is therefore opposed by capillary π_c_ [i.e., osmotic suction: (P_c_ - π_c_)] and Pi is opposed by π_i_ (Pi - π_i_). The Starling hypothesis asserts that fluid is filtered at the arterial end of the capillary because Pc predominates over all other forces, and that fluid is reabsorbed at the venous end of the capillary because π_c_ (osmotic suction) predominates. Interstitial forces (Pi, π_i_) act as modulators of the rate of fluid flux and therefore the volume of Jv (14, 185). Later studies modified Starling's hypothesis to account for transvascular fluid flux rates per unit pressure (i.e., hydraulic conductance: L_p_) and the macromolecular sieving properties of the microvascular barrier (Staverman's reflection coefficient: σ) [(12–14); Woodcock and Michel; (173, 187)]. Both L_p_ and σ vary among different types of capillaries since L_*p*_ is dependent upon the number of “pores” and σ is dependent on effective pore diameter. The σ for most plasma solutes ranges from 0 to 1 (i.e., 0 = totally permeable; 1 = totally impermeable) (187). The capillary wall osmotic and σ for water, anions, cations, and smaller soluble substances like glucose is nearly 0 (freely permeable) (160). Larger plasma solutes (>30–40 kDa), like albumin (66–69 kDa; diameter ~3.5 nm), which accounts for 80% of total plasma protein and commercial semisynthetic colloid solutions (i.e., gelatins, dextran, and hydroxyethyl starches; COP range 24–60 mm Hg) exhibit σ's ranging from 0.7 to 1.0 and are almost impermeant to most the microvascular barrier except the sinusoids of the liver. The incorporation of L_p_ and σ into Starlings hypothesis is the basis for what is proclaimed as the “Starling equation” that is still published in most texts [J_v_ = L_*p*_ [(P_c_ – P_i_) – σ (π_c_ – π_i_)]], although Starling had little to do with its derivation since the earliest form of the equation did not appear until 1927 (182).

### Contemporary Theory

Recent investigations have led to a revision of the Starling hypothesis (165) and the Starling equation based upon GLX COP (π_g_): J_v_ = L_p_ [(P_c_ – P_i_) – σ (π_c_ – π_g_)] [(11); Woodcock and Michel; (188–193)]. It is now realized that the interstitial COP does not directly determine fluid movement across the microvascular wall, and that the effect of π_c_ on J_v_ is far less than originally predicted (11, 189–195). The sieving properties of the glycocalyx modify Starling's forces by imposing an obstacle to J_v._ The π difference across non-fenestrated capillaries is influenced by the π_g_ and π_i_ is far less important in determining Jv than originally proposed. Notably, π_g_ is negligible compared to π_c_ such that the osmotic pressure gradient across the glycocalyx is close to π_c_ rather than the difference between π_c_ and π_i_. Fluid that is filtered through the glycocalyx flows rapidly through narrow inter-endothelial cell breaks, thereby limiting interstitial protein back diffusion into the sub-glycocalyx space. The “Revised” Starling equation [(11); Woodcock and Michel; (189)] has proven to be more consistent with experimental and clinical observations and suggests that (1) J_v_ is far less than originally predicted; (2) Fluid is not normally reabsorbed from the venous end of the capillary during normal physiologic conditions (steady state no-reabsorption rule); (3) Tissue lymph drainage is the primary route for return of interstitial fluid to the circulation; (4) Interstitial fluid is reabsorbed from the interstitium when P_c_ decreases until a new steady state is established (14); and (5) Crystalloid is almost as effective as a colloid (Col) administration for treating hypovolemia from blood loss (11, 173–176). These revisions highlight the importance of GLX composition and integrity and the number of inter-endothelial cellular “breaks” (i.e., glycocalyx-junction-break model) in determining the effectiveness of fluid resuscitation (195). They do not negate the “importance of transcapillary refill” as suggested by some (196), but do have important implications regarding fluid selection, rate, and volume for improving fluid efficiency and effectiveness in diseased animals [Woodcock and Michel; (189, 194, 197)].

## Volume Kinetics

Volume kinetics (VK) determines the volume into which an administered fluid is distributed (i.e., volume of distribution: V_d_), the volume of plasma that is completely cleared of the administered fluid per unit time (i.e., clearance: Cl) and the time it takes for the total amount of administered fluid to be reduced by one-half of its original volume (i.e., half-life: t_1/2_) (31). Intravenous fluids are initially distributed into a central compartment (V_c_) followed by diffusion into a peripheral compartment (V_t_) [(31); Yiew, Bateman, Hahn, Bersenas, Muir; (179, 198–201)]. The distribution half-time for most crystalloids is relatively short (<8–10 min) implying that distribution is complete within ~30–50 min (4–5 half-lives), a range that closely coincides with the measured half-lives reported for acetated (56 min) and lactated (50 min) Ringer's solutions in humans (155). A low Cl_d_ from V_c_ increases the infused fluid's potency (i.e., the volume required to expand the plasma volume by 20% in 30 min) but also increases hemodilution. The Cl_d_ for colloidal solutions [i.e., hydroxyethyl starches (HES)] is much lower than crystalloids, suggesting delayed departure from V_c_ and prolongation of their volume expanding effects.

Rapid fluid administration rates (>40–60 ml/kg/hr) and large fluid volumes (>60–80 ml/kg) produce hemodilution, interstitial fluid accumulation (i.e., edema), and serious rebleeding in animals with uncontrolled hemorrhage (15, 78, 83, 202, 203). Most anesthetic drugs, particularly inhalant anesthetics (e.g., propofol, isoflurane), depress cardiorespiratory function, blunt homeostatic reflexes, promote vasoplegia, [Valverde; (204–206)] decrease tolerance to acute anemia [i.e., increase the critical Hb concentration: (Hb_crit_)] (207, 208), promote interstitial fluid accumulation (209) and perioperative fluid retention (209–212), decrease urine output (212, 213), and depress the response to fluid administration (204, 214). In addition, vasoactive drugs are known to alter fluid volume kinetics (215–219). Stimulation of alpha1- adrenergic receptors (e.g., norepinephrine; phenylephrine) increases V_d_, Cl_d_, the accumulation of fluid in V_t_, and Cl_r_ while stimulation of beta-1 adrenergic receptors (e.g., isoproterenol) increase V_c_ and decrease V_d_, Cl_d_, and Cl_r_ (69, 216, 217, 220). Notably, fluid accumulation in V_t_ is more significantly influenced by the rate of infusion (i.e., ml/kg/min) than by the infused fluid volume; higher infusion rates produce greater degrees of interstitial fluid accumulation, hemodilution, coagulation abnormalities, and organ dysfunction (79, 199, 203, 221, 222).

## New Horizons

New fluids and goal directed fluid therapies (GDFT) continue to be developed for the treatment of specific naturally occurring diseases with the goals of improving tissue oxygenation and perfusion [(9); Edwards and Hoareau; (197, 223–228)], and reducing adverse events and mortality (229, 230). Damage control resuscitation (DCR) strategies limit the amount of crystalloids administered and employ balanced blood product resuscitation ratios [PRBC's-plasma-platelets ratio of 1:1:1; Hall and Drobatz; Boysen and Gommeren; (230–235)]. Isotonic and hypertonic crystalloid solutions continue to be investigated in order to rapidly restore hemodynamics, reduce the amount of fluid administered in order minimize hemodilution, and tissue edema, and lessen the development of disseminated intravascular coagulation (58–62, 236, 237). Novel therapies that mimic natural hemostatic mechanisms (68) or reduce vascular leakage (238–240) are being developed and solutions that increase tissue oxygenation (e.g., hemoglobin) and restore microcirculatory blood flow continue to evolve (241–243). Future fluids should protect or repair the endothelium (224, 228, 238, 244, 245). Methods for determining their success will be dependent upon the development of validated dynamic non or minimally invasive hemodynamic monitoring methodologies [(42); Cooper and Silverstein; Boysen and Gommeren; (20, 38–41, 235, 246–254)] in addition assessment of thromboelastographic variables (249), implementation of deep-learning algorithms (254) and development of bio-responsive drug delivery systems [Gholami et al.; (255–260)]. It is hoped that the information contained within this compendium will inspire readers to employ fluid therapy practices that improve patient outcome.

## Author Contributions

All authors listed have made a substantial, direct and intellectual contribution to the work, and approved it for publication.

## Conflict of Interest

The authors declare that the research was conducted in the absence of any commercial or financial relationships that could be construed as a potential conflict of interest.

## Publisher's Note

All claims expressed in this article are solely those of the authors and do not necessarily represent those of their affiliated organizations, or those of the publisher, the editors and the reviewers. Any product that may be evaluated in this article, or claim that may be made by its manufacturer, is not guaranteed or endorsed by the publisher.
